# Agreement and diagnostic performance of a smartphone-based bilirubin assessment tool for screening neonatal hyperbilirubinemia

**DOI:** 10.3389/fped.2026.1852961

**Published:** 2026-06-19

**Authors:** Ya Zhao, Anlan Xu, Tonghui Suo, Fengqin Zhang, Mingwu Chen

**Affiliations:** 1 Department of Pediatrics, Anhui Provincial Hospital, Affiliated Hospital of Wannan Medical University, Hefei, Anhui, China; 2 Division of Life Sciences and Medicine, Department of Pediatrics, The First Affiliated Hospital of USTC, University of Science and Technology of China, Hefei, Anhui, China; 3 Division of Life Sciences and Medicine, Department of Obstetrics and Gynecology, The First Affiliated Hospital of USTC, University of Science and Technology of China, Hefei, Anhui, China

**Keywords:** diagnostic screening, neonatal hyperbilirubinemia, neonatal jaundice, smartphone application, transcutaneous bilirubin

## Abstract

**Background:**

Neonatal hyperbilirubinemia is common and may lead to severe neurological injury if not detected and treated promptly. Total serum bilirubin (TSB) remains the reference standard, while transcutaneous bilirubin (TcB) is commonly used for non-invasive screening. Smartphone-based bilirubin assessment may provide a supplementary approach for neonatal jaundice screening.

**Objective:**

This study aimed to evaluate the agreement, correlation, and diagnostic performance of a self-developed mobile application for bilirubin measurement compared with TSB and TcB in screening neonatal hyperbilirubinemia.

**Methods:**

This prospective diagnostic study was conducted in the neonatal department of Anhui Provincial Hospital, China, between March 2025 and January 2026. Eligible neonates undergoing clinically indicated TSB testing for jaundice before phototherapy were included. TSB, TcB, and mobile app–derived bilirubin measurements were obtained during the same assessment session. Using TSB as the reference standard, agreement between mobile app–derived bilirubin and TSB was assessed using Bland–Altman analysis, and correlation was evaluated using linear regression. Multivariable logistic regression models adjusted for gestational age, postnatal age, and birth weight were used to evaluate diagnostic performance, and receiver operating characteristic curves were generated from predicted probabilities.

**Results:**

Among 121 neonates, 46 were preterm and 75 were term infants. In the overall cohort, mobile app–derived bilirubin did not differ significantly from TSB, with a mean difference of −2.42 μmol/L and 95% limits of agreement from −30.57 to 25.73 μmol/L. Agreement was lower among preterm infants than among term infants. Mobile app–derived bilirubin was positively correlated with TSB (*R*^2^ = 0.776, *P* < 0.001). In the adjusted logistic regression model, mobile app–derived bilirubin was independently associated with neonatal hyperbilirubinemia (odds ratio = 1.047, 95% confidence interval: 1.019–1.081, *P* = 0.002). The mobile application achieved an area under the curve of 0.936, with sensitivity of 0.912 and specificity of 0.908, while TcB achieved an area under the curve of 0.937, with sensitivity of 0.882 and specificity of 0.931.

**Conclusion:**

Mobile application–derived bilirubin showed good agreement with TSB and diagnostic performance comparable to TcB under standardized conditions. Owing to its non-invasive, convenient, and low-cost nature, this smartphone-assisted approach has potential as a supplementary tool for neonatal hyperbilirubinemia screening, pending further technical optimization and multicenter home-based validation.

## 1 Introduction

Neonatal jaundice is common worldwide, affecting approximately 60% of term infants and 80% of preterm infants during the first week of life (1, 2). Neonatal jaundice is characterized by yellow discoloration of the skin, mucous membranes, and sclera caused by elevated bilirubin levels (3). Owing to immature bilirubin metabolism in neonates, bilirubin may accumulate and progress to hyperbilirubinemia if not recognized and managed appropriately (4). Although most cases are physiological and self-limited, severe hyperbilirubinemia can lead to bilirubin encephalopathy and long-term neurological impairment (5).

Total serum bilirubin (TSB) and transcutaneous bilirubin (TcB) are the main methods currently used for bilirubin assessment. TSB remains the reference standard but requires blood sampling, which may increase discomfort in neonates. TcB is non-invasive and suitable for repeated monitoring (6, 7), but it generally requires dedicated equipment and is mainly used in clinical settings. Therefore, accessible and non-invasive approaches that can support bilirubin screening beyond hospital settings are needed, particularly for post-discharge follow-up and resource-limited areas.

In recent years, smartphone-based monitoring technologies and multimodal data processing methods have shown potential in medical assessment and remote health monitoring (8–10). Several studies have explored machine learning, artificial intelligence, or multimodal approaches for neonatal jaundice assessment (11, 12). However, the clinical performance and generalizability of such approaches require further validation against established bilirubin measurement methods.

This study aimed to evaluate the agreement and correlation between bilirubin values obtained using a self-developed mobile application and TSB, and to compare the diagnostic performance of the mobile application with TcB for screening neonatal hyperbilirubinemia.

## 2 Materials and methods

### 2.1 Study population

This prospective diagnostic study enrolled neonates admitted to the neonatal ward of Anhui Provincial Hospital, China, between March 2025 and January 2026. The inclusion criteria were as follows: gestational age between 29⁺⁴ and 41 weeks, postnatal age ≤28 days, and clinically indicated TSB testing for jaundice before phototherapy. The exclusion criteria were severe skin infection or ulceration at the measurement site, capillary refill time >5 s, transcutaneous oxygen saturation <85%, or refusal of informed consent by the guardians. Neonates were withdrawn from the study if they left the hospital before completing the required assessment, developed severe complications that prevented further evaluation, or had incomplete key measurement data. The study was approved by the Ethics Committee of Anhui Provincial Hospital (approval No. 2022KY Ethics Review No. 251). Written informed consent was obtained from the guardians of all enrolled neonates.

### 2.2 Sample size estimation

This diagnostic study used TSB as the reference standard to evaluate the screening performance of the mobile application and TcB. Based on previous studies reporting high diagnostic performance for smartphone-based bilirubin assessment, the expected area under the receiver operating characteristic curve was set at 0.90 (13). PASS software was used for sample size estimation, with a null hypothesis area under the curve of 0.75, *α* = 0.05, and power = 0.90. The minimum required sample size was 58. Considering subgroup analysis and potential data loss, 121 neonates were ultimately included.

### 2.3 Data collection

Clinical data were collected from electronic medical records, including gestational age, postnatal age, sex, birth weight, cephalohematoma, family history, and bilirubin measurement results. The bilirubin measurements included TSB, TcB, and mobile app–derived bilirubin values. All measurements used for analysis were obtained before phototherapy.

### 2.4 Mobile application and bilirubin measurement system

The mobile application used in this study was developed for neonatal jaundice screening based on smartphone skin image analysis and color calibration. The system included a mobile terminal, image acquisition module, color calibration card, bilirubin estimation module, and data storage function. The bilirubin estimation algorithm generated an estimated bilirubin value from neonatal skin images acquired with a standard color calibration card. The software copyright registration number is 2025SR0221435.

The output value generated by the application was defined as mobile app–derived bilirubin. The present study focused on the clinical validation of mobile app–derived bilirubin under standardized hospital-based measurement conditions. Cloud-based data upload, remote monitoring, automated alerts, and model updating functions were part of the system design but were not independently evaluated in this study.

### 2.5 Measurement procedure and diagnostic definition

TSB, TcB, and mobile app–derived bilirubin measurements were obtained during the same assessment session before phototherapy. TcB and mobile app–derived bilirubin were each measured three times, and the mean value was used for final analysis. The skin measurement area was kept clean and dry, without grease, ointment, covering material, bruising, edema, or obvious skin lesions.

The diagnosis of neonatal hyperbilirubinemia was determined according to the hour-specific bilirubin nomogram recommended by the Guidelines for the Diagnosis and Treatment of Neonatal Hyperbilirubinemia. Gestational age and postnatal age were considered when determining the bilirubin risk threshold. A bilirubin level exceeding the 95th percentile was defined as neonatal hyperbilirubinemia for subsequent agreement, correlation, regression, and diagnostic performance analyses.

### 2.6 Standardized operating procedure for mobile app–derived bilirubin measurement

To improve the accuracy and repeatability of image acquisition, a standardized operating procedure was used. Measurements were performed under natural daylight or soft indoor diffuse lighting. Direct sunlight, strong light sources, backlighting, obvious shadows, and glare were avoided. The smartphone camera was positioned perpendicular to the measurement site, and the distance between the smartphone and the skin was maintained at approximately 30 cm.

The color calibration card was placed adjacent to the measurement site and fully included within the image frame. The application used the color information from the calibration card to reduce the influence of ambient lighting and camera-related color variation. Images were reacquired if the color card was incomplete, blurred, obstructed, contaminated, faded, or affected by glare or shadow. For each measurement, three consecutive images were obtained, and the average bilirubin value generated by the application was recorded as the final mobile app–derived bilirubin value.

### 2.7 Statistical analysis

Continuous variables were expressed as mean ± standard deviation, and categorical variables were expressed as number and percentage. Mean differences between TSB and mobile app–derived bilirubin were assessed using a paired *t*-test. Agreement between TSB and mobile app–derived bilirubin was evaluated using Bland–Altman analysis, with the mean difference and 95% limits of agreement calculated. Correlation was assessed using linear regression, and the coefficient of determination was reported.

Neonatal hyperbilirubinemia, defined according to the hour-specific bilirubin nomogram, was used as the dependent variable in multivariable logistic regression analyses. Two models were constructed separately. In the mobile application model, mobile app–derived bilirubin was the main independent variable. In the TcB model, TcB was the main independent variable. Both models were adjusted for gestational age, postnatal age, and birth weight. Odds ratios, 95% confidence intervals, and *P* values were calculated. Model calibration was assessed using the Hosmer–Lemeshow goodness-of-fit test. Receiver operating characteristic curves were generated based on the predicted probabilities from the logistic regression models. The area under the curve, 95% confidence interval, sensitivity, and specificity were calculated to evaluate diagnostic performance. All statistical analyses were performed using R software, version 4.3.2 (R Foundation for Statistical Computing, Vienna, Austria). A two-sided *P* value <0.05 was considered statistically significant.

## 3 Results

### 3.1 Patient characteristics

A total of 121 neonates with jaundice were included in this study. Among them, 62 were male (51.2%) and 59 were female (48.8%). Forty-six neonates were preterm infants (<37 weeks, 38.0%), and 75 were term infants (≥37 weeks, 62.0%). The mean birth weight was 2,830.50 ± 675.35 g. The mean TSB, TcB, and mobile app–derived bilirubin values were 154.35 ± 29.58 μmol/L, 160.07 ± 26.88 μmol/L, and 156.77 ± 22.85 μmol/L, respectively (Table 1).

**Table 1:** Baseline clinical characteristics and bilirubin measurements of neonates included in the prospective diagnostic study.

Characteristic	Value
Total neonates, *n*	121
Birth weight, g	2,830.50 ± 675.35
Sex, *n* (%)	
Male	62 (51.20)
Female	59 (48.80)
Gestational age group, *n* (%)	
Preterm (<37 weeks)	46 (38.00)
Term (≥37 weeks)	75 (62.00)
TSB, μmol/L	154.35 ± 29.58
TcB, μmol/L	160.07 ± 26.88
Mobile app-derived bilirubin, μmol/L	156.77 ± 22.85

Data are presented as mean ± standard deviation or *n* (%). TSB, total serum bilirubin; TcB, transcutaneous bilirubin.

### 3.2 Agreement between mobile app–derived bilirubin and TSB

**Figure 1:**
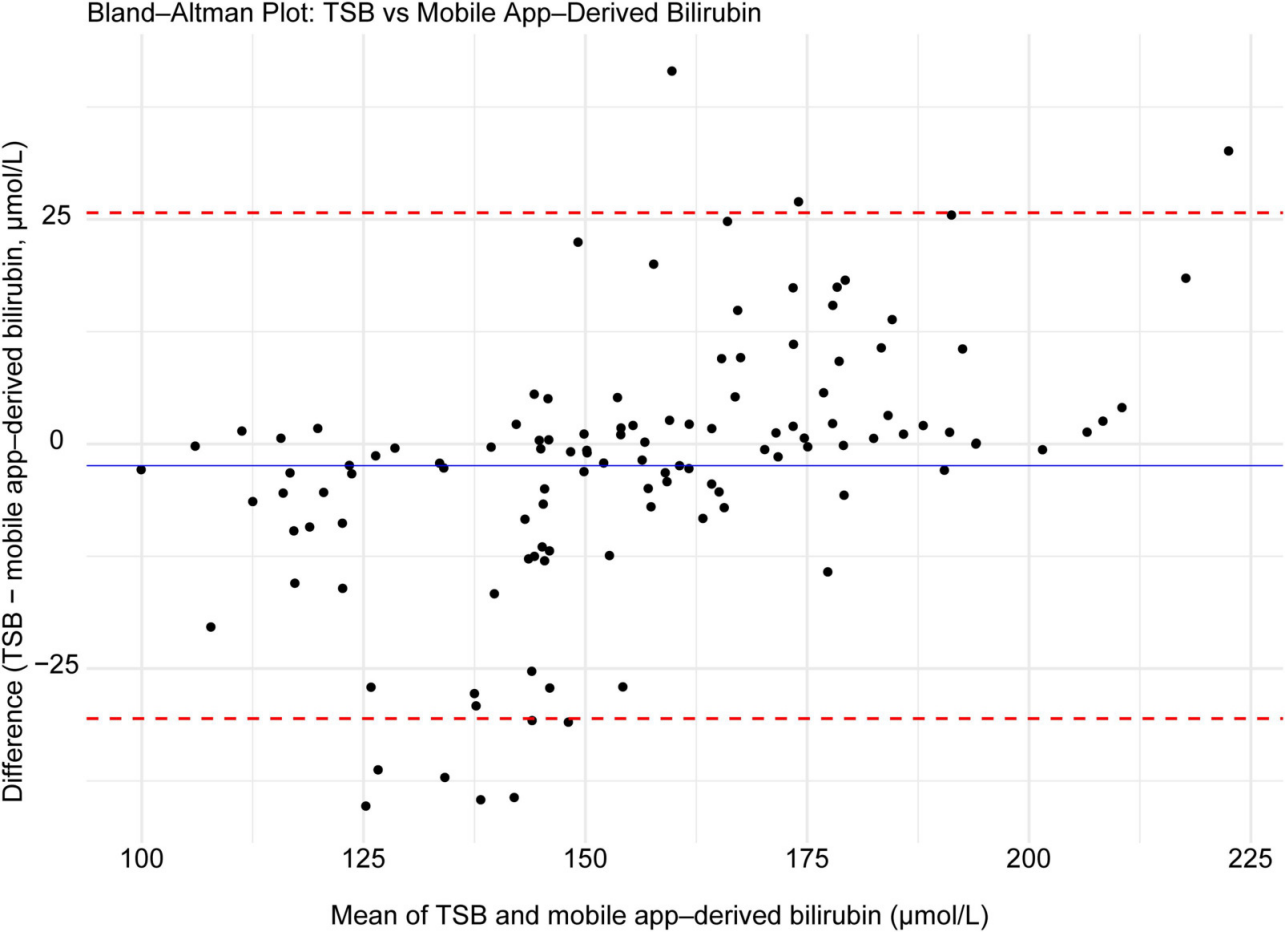
Bland–Altman plot showing agreement between mobile app–derived bilirubin and total serum bilirubin in neonates with jaundice. The *x*-axis represents the mean of TSB and mobile app–derived bilirubin, and the *y*-axis represents the difference between TSB and mobile app–derived bilirubin. The solid line indicates the mean difference, and the dashed lines indicate the 95% limits of agreement.

In the overall cohort, the mean difference between TSB and mobile app–derived bilirubin was −2.42 μmol/L. The difference was not statistically significant (*t* = −1.85, *P* = 0.07). Bland–Altman analysis showed 95% limits of agreement from −30.57 to 25.73 μmol/L (Table 2 and Figure 1).

**Table 2:** Agreement between mobile app–derived bilirubin and total serum bilirubin in the overall cohort and by gestational age group.

Group	*n*	Mean difference, μmol/L	*t* value	*P* value	Lower 95% LoA, μmol/L	Upper 95% LoA, μmol/L
Overall cohort	121	−2.42	−1.85	0.07	−30.57	25.73
Preterm infants	46	−5.20	−2.48	0.02	−33.00	22.60
Term infants	75	−0.71	−0.43	0.67	−28.73	27.31

Mean difference was calculated as TSB minus mobile app–derived bilirubin. LoA, limits of agreement; TSB, total serum bilirubin.

In subgroup analysis, the mean difference was −5.20 μmol/L in preterm infants, with 95% limits of agreement from −33.00 to 22.60 μmol/L. The difference between TSB and mobile app–derived bilirubin was statistically significant in the preterm group (*t* = −2.48, *P* = 0.02). In term infants, the mean difference was −0.71 μmol/L, with 95% limits of agreement from −28.73 to 27.31 μmol/L, and no significant difference was observed between the two methods (*t* = −0.43, *P* = 0.67) (Table 2).

### 3.3 Correlation between mobile app–derived bilirubin and TSB

**Figure 2:**
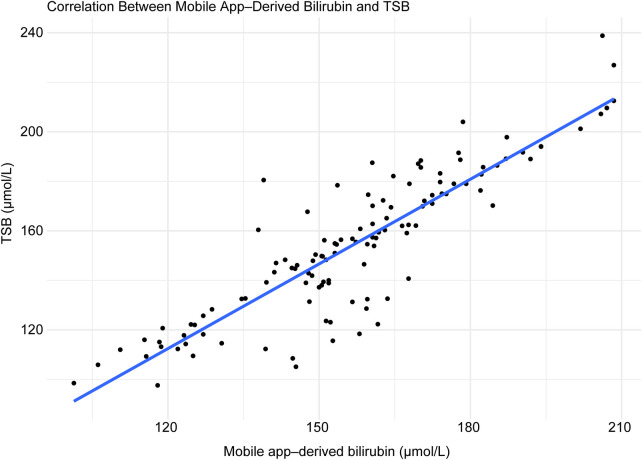
Linear association between mobile app–derived bilirubin and total serum bilirubin in neonates with jaundice. The solid line represents the fitted line from linear regression. The coefficient of determination was *R*^2^ = 0.776, indicating that mobile app–derived bilirubin explained 77.6% of the variance in total serum bilirubin. *P* < .001 for the regression slope.

Linear regression analysis showed a positive correlation between mobile app–derived bilirubin and TSB in the overall cohort. The coefficient of determination was *R*^2^ = 0.776 (*P* < 0.001), indicating that mobile app–derived bilirubin explained 77.6% of the variation in TSB values (Figure 2).

### 3.4 Multivariable logistic regression analysis

Multivariable logistic regression was performed to evaluate factors associated with neonatal hyperbilirubinemia. In the mobile application model, mobile app–derived bilirubin was independently associated with neonatal hyperbilirubinemia after adjustment for gestational age, postnatal age, and birth weight (odds ratio [OR] = 1.047, 95% confidence interval [CI]: 1.019–1.081, *P* = 0.002).

In the TcB model, TcB was independently associated with neonatal hyperbilirubinemia (OR = 1.042, 95% CI: 1.018–1.071, *P* = 0.001). The Hosmer–Lemeshow test indicated acceptable calibration for both models, with *χ*^2^ = 13.857, df = 8, *P* = 0.086 for the mobile application model and *χ*^2^ = 10.777, df = 8, *P* = 0.215 for the TcB model (Table 3).

**Table 3:** Adjusted logistic regression models for the association of mobile app–derived bilirubin and transcutaneous bilirubin with neonatal hyperbilirubinemia.

Model	Variable	OR	95% CI	*P* value
Mobile application model	Mobile app–derived bilirubin	1.047	1.019–1.081	0.002
	Gestational age	0.772	0.467–1.201	0.271
	Postnatal age	0.308	0.172–0.473	<0.001
	Birth weight	1.001	1.000–1.003	0.159
TcB model	TcB	1.042	1.018–1.071	0.001
	Gestational age	0.765	0.454–1.200	0.268
	Postnatal age	0.311	0.178–0.473	<0.001
	Birth weight	1.001	1.000–1.003	0.148

The dependent variable was neonatal hyperbilirubinemia. Hosmer–Lemeshow goodness-of-fit test: mobile application model, *χ*^2^ = 13.857, df = 8, *P* = 0.086; TcB model, *χ*^2^ = 10.777, df = 8, *P* = 0.215. OR, odds ratio; CI, confidence interval; TcB, transcutaneous bilirubin.

### 3.5 Diagnostic performance

**Figure 3:**
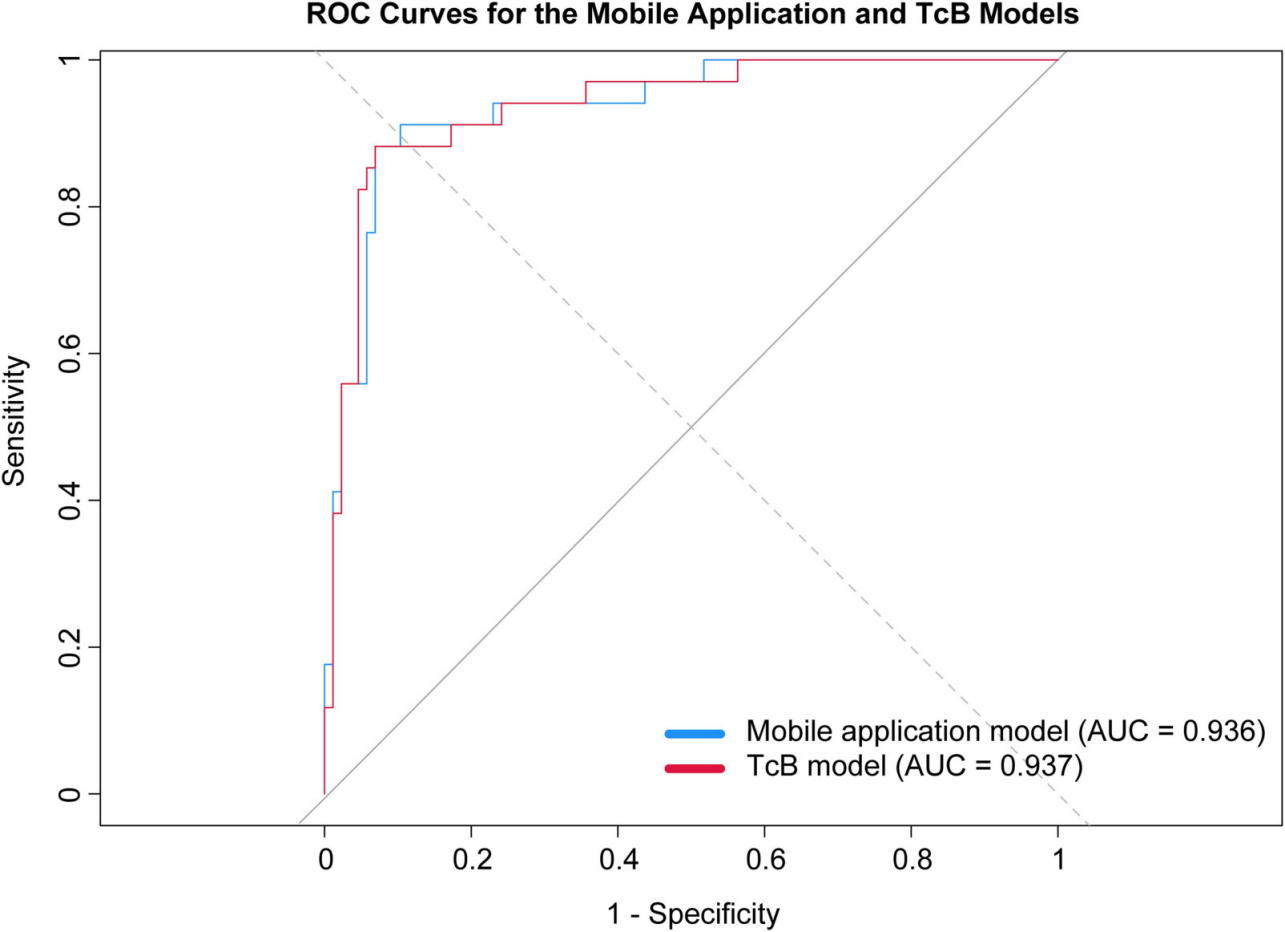
Receiver operating characteristic curves for adjusted logistic regression models based on mobile app–derived bilirubin and transcutaneous bilirubin for neonatal hyperbilirubinemia screening. Neonatal hyperbilirubinemia was defined according to the hour-specific bilirubin nomogram. Receiver operating characteristic curves were generated using predicted probabilities from logistic regression models adjusted for gestational age, postnatal age, and birth weight. AUC indicates area under the receiver operating characteristic curve; TcB, transcutaneous bilirubin.

Using neonatal hyperbilirubinemia defined according to the hour-specific bilirubin nomogram as the outcome, receiver operating characteristic curves were generated based on the predicted probabilities from the adjusted logistic regression models. The mobile application model achieved an area under the curve (AUC) of 0.936 (95% CI: 0.889–0.984), with a sensitivity of 0.912 and specificity of 0.908. The TcB model achieved an AUC of 0.937 (95% CI: 0.890–0.985), with a sensitivity of 0.882 and specificity of 0.931 (Table 4 and Figure 3).

**Table 4:** Diagnostic performance of adjusted logistic regression models based on mobile app–derived bilirubin and transcutaneous bilirubin for neonatal hyperbilirubinemia screening.

Method	AUC (95% CI)	Sensitivity	Specificity
Mobile application model	0.936 (0.889–0.984)	0.912	0.908
TcB model	0.937 (0.890–0.985)	0.882	0.931

Receiver operating characteristic analysis was performed using predicted probabilities from logistic regression models adjusted for gestational age, postnatal age, and birth weight. AUC, area under the curve; CI, confidence interval; TcB, transcutaneous bilirubin.

## 4 Discussion

This prospective diagnostic study found that mobile app–derived bilirubin showed measurable agreement and significant correlation with TSB in neonates with jaundice. Its adjusted screening performance for neonatal hyperbilirubinemia was close to that of TcB, suggesting that smartphone-assisted bilirubin assessment may provide supplementary information for clinical screening when image acquisition is standardized.

Timely bilirubin monitoring is essential because severe or persistent hyperbilirubinemia can lead to bilirubin encephalopathy and long-term neurological sequelae (14–16). In this context, smartphone-based and portable optical or imaging technologies have attracted increasing attention for non-invasive bilirubin or related biomarker assessment (17–20). The present study adds to this field by comparing mobile app–derived bilirubin not only with TSB, the reference standard, but also with TcB, a commonly used non-invasive method.

The agreement analysis showed that mobile app–derived bilirubin was broadly consistent with TSB in the overall cohort, but agreement was weaker in preterm infants. This finding is important because correlation alone is insufficient to judge whether a new measurement method can be used in clinical practice. Previous studies have also reported promising performance of smartphone-based bilirubin assessment. Ngeow et al. found a strong correlation between mobile application–predicted bilirubin values and TSB (13), and a meta-analysis by Hegde et al. reported favorable accuracy of smartphone applications, particularly at low-to-moderate bilirubin levels (21). Our findings are consistent with these reports, while further indicating that gestational age may influence measurement stability.

Diagnostic studies of non-invasive jaundice assessment commonly evaluate threshold-related performance, including sensitivity, specificity, and ROC-based accuracy (22, 23). In the present study, diagnostic performance was evaluated using a clinically relevant risk definition rather than a single fixed bilirubin cutoff. Neonatal hyperbilirubinemia was defined according to the hour-specific bilirubin nomogram recommended by current guidelines, with the 95th percentile used as the threshold (24). This approach reflects the fact that bilirubin risk is influenced by postnatal age and gestational age. The adjusted ROC analysis showed that the mobile application model achieved an AUC close to that of the TcB model, supporting its potential value as a supplementary screening approach.

From a clinical perspective, smartphone-assisted bilirubin assessment may be useful as a convenient and low-cost adjunct to existing screening methods, especially in settings where access to dedicated bilirubinometry equipment is limited (25). However, its role should be considered complementary rather than substitutive. The present results were obtained under standardized hospital-based conditions, and they do not establish the accuracy of unsupervised home use.

The lower agreement observed in preterm infants may be related to differences in skin optical properties, bilirubin kinetics, and clinical vulnerability compared with term infants. Previous studies have suggested that bilirubin assessment in preterm or clinically vulnerable neonates may be influenced by gestational age, optical measurement conditions, and underlying etiologies of jaundice (26–30). These factors may reduce the stability of image-based bilirubin estimation and indicate that serum bilirubin confirmation remains important when assessing preterm infants or clinically unstable neonates.

This study has several limitations. First, it was conducted at a single center with a modest sample size, which may limit generalizability. Second, the number of neonates with severe hyperbilirubinemia was limited, and performance at higher bilirubin levels requires further evaluation. Third, subgroup analysis was limited to preterm vs. term infants, without more detailed stratification by gestational age. Fourth, all measurements were performed under controlled conditions; in home settings, lighting, camera quality, shooting angle, distance, and caregiver operation may affect image quality and measurement accuracy. Finally, remote monitoring, automated alerts, and long-term follow-up functions of the application were not independently validated in this study.

## 5 Conclusion

Mobile app-derived bilirubin demonstrated acceptable agreement with TSB and adjusted screening performance close to that of TcB in this single-center cohort. This smartphone-assisted approach may provide a convenient, non-invasive, and low-cost supplementary option for neonatal hyperbilirubinemia screening, but further technical optimization, multicenter validation, and home-based testing are warranted.

## Data Availability

The original contributions presented in the study are included in the article/Supplementary Material, further inquiries can be directed to the corresponding author.
